# Pulse pressure variation shows a direct linear correlation with tidal volume in anesthetized healthy patients

**DOI:** 10.1186/s12871-016-0233-y

**Published:** 2016-09-08

**Authors:** Yi Liu, Jing-sheng Lou, Wei-dong Mi, Wei-xiu Yuan, Qiang Fu, Min Wang, Jing Qu

**Affiliations:** 1 Anesthesiology and Operation Center, Chinese PLA General Hospital, 28 Fuxing Road, Haidian District, Beijing, 100853 China; 2 Department of Anesthesiology, Beijing Tongren hospital, Capital Medical University, Beijing, 100041 China; 3 Graduate School, Xuzhou Medical College, Xuzhou, 221004 Jiangsu Province China

**Keywords:** Pulse pressure variation, Tidal volume, Blood pressure, Volume, Mechanical ventilation

## Abstract

**Background:**

The settings of mechanical ventilation, like tidal volume (VT), occasionally need to be adjusted in the process of anesthesia for some special reasons. The aim of this study was therefore to assess the relationship between pulse pressure variations (PPVs) in different settings of VT in anesthetized healthy patients under mechanical ventilation.

**Methods:**

Sixty nine ASA I-II patients scheduled for gastrointestinal surgery under general anesthesia were included in this prospective study. All the patients were ventilated at a VT of 6, 8 or 10 ml/kg (predicted body weight) with no positive end expiratory pressure (PEEP) in a random order after intubation. PPV, mean arterial blood pressure, and other hemodynamic and respiratory parameters were recorded in each VT setting respectively after Partial Pressure of End-Tidal Expiration Carbon Dioxide (PetCO_2_) maintained between 30 mmHg and 40 mmHg by changing Respiratory Rate (RR) before incision.

**Results:**

The values of PPV at different settings of VT showed a tight correlation between each other (6 vs. 8 ml/kg: *r* = 0.97, *P* < 0.0001; 6 vs.10 ml/kg: *r* = 0.95, *P* < 0.0001; 8 vs. 10 ml/kg: *r* = 0.98, *P* < 0.0001, respectively).

**Conclusion:**

There is a direct linear correlation between PPVs at different tidal volumes in anesthetized ASA I-II patients. PPV in any of the 3 VT settings (6, 8 or 10 ml/kg) can deduce that in any other 2 settings. Further studies are needed to explore the effect of intraoperative confounders for this knowledge to be clinically applied.

**Trial registration:**

NCT01950949, www.clinicaltrials.gov, July 26, 2013.

## Background

Adequate fluid administration can increase cardiac output, improve microcirculatory perfusion, avoid tissue edema and shorten hospital stay in patients [1–4]. Pulse pressure variation has recently been recommended as an effective predictor of fluid responsiveness in patients receiving mechanical ventilation [5, 6]. Previous studies have demonstrated the excellent predictive power of PPV on fluid responsiveness just at a VT higher than 8 ml/kg, the predictive accuracy of PPV at a low VT remains uncertain [7–9]. In addition, a series of studies suggested to carry out the lung-protective ventilation strategies at a low VT and low positive end-expiratory pressure both in critically ill and patients undergoing elective operation [10–12]. Similar to stroke volume variation (SVV), PPV value could increase proportionally in the same cardiovascular state in anesthetized mongrel dogs as VT increases [13]. And there is a significant linear correlation between PPVs at a VT of 6 and 8 ml/kg in patients with severe sepsis and septic shock after resuscitation [14]. However, there have been few studies revealing the actual relationship between PPVs in various different VT settings in patients without cardiopulmonary diseases. Sometimes, in order to maintain the appropriate PetCO_2_ level in some special operations (laparoscopic surgery, robotic surgery, cerebral surgery, etc.), ventilator settings, especially the VT in the volume-controlled ventilation, vary during the course of a surgical procedure [15]. Therefore, the objective of this study was to examine the relationship between the values of PPV under different VT settings in anesthetized patients without cardiopulmonary disorder.

## Methods

### Patients

This study was approved by the Ethical Committee of the Chinese PLA General Hospital (Registration No. 20111124-028, Chinese PLA General Hospital, Beijing 100853, China, chairperson Kun-lun He) and was registered at www.clinicaltrials.gov (ID: NCT01950949). Informed written consent was obtained from each patient. Adult patients (aged 18–60 years, ASA I or II) scheduled for elective gastrointestinal surgery (open or laparoscopic approach) were enrolled prospectively between July 2013 and March 2014. Patients with suspected acute respiratory distress syndrome, cardiovascular diseases, neurological disease, liver and renal function abnormal, or diabetes mellitus, and those whose predicted body weight did not match actual body weight were excluded. All the patients received oral sodium phosphate solution the night before the surgery prescribed by the surgeons for gastrointestinal preparation.

### Procedure

Once in the operating room, an infusion of Lactated Ringer's solution was started at 4 ml.kg^−1^.h^−1^ through the 18G upper limb venous trocar. Anesthesia was induced using midazolam (0.04 mg.kg^−1^), fentanyl (3 μg.kg^−1^), propofol (1.5–2.5 mg.kg^−1^) and rocuronium (0.6 mg.kg^−1^), and maintained by an intravenous infusion of propofol to keep the Bispectral index (Aspect Bis Monitor XP) between 40 and 60. After tracheal intubation, a 20 G artery catheter was inserted into the left radial artery and connected to a bed-side monitor (Philips Intellivue MP50). Pressure transducers were zeroed at the mid-axillary level to atmospheric pressure. All patients were ventilated at a VT of 6, 8 or 10 ml/kg (predicted body weight) with 50 % oxygen with air without PEEP in a random order and successively for 3 min in each setting. During this period, the respiratory rate was adjusted to maintain an PetCO_2_ between 30 and 40 mmHg. PPV, arterial blood pressure, heart rate (HR), peak airway pressure (Ppeak), mean airway pressure (Pmean), RR, PetCO_2_ and BIS value were recorded 3 min after VT change while the vital signs were stable. Then the parameters under the three settings of VTs were recorded. In the case that PPV value was larger than 13 % at a VT of 8 ml/kg, 10 ml/kg of 6 % hydroxyethyl starch solution (HES 130/0.4; VOLUVEN; Fresenius Kabi, Stans, Switzerland) would be given to these patients (defined as subgroup) for volume expansion over 15 min. And then the next round of recording was conducted in a new cycling of these three VT settings. All the parameters were obtained before skin incision in the supine position. During these periods, 5 mg ephedrine was administered when SAP lowered below 90 mmHg, and this case would be eliminated.

### Statistical analysis

Statistical analyses were performed using SPSS 13.0 for Windows (SPSS, Chicago, IL, USA). Normally distributed data were presented as mean ± standard deviation. Non-normally distributed data were presented as median (25–75 % interquartile range). Hemodynamic data and respiratory parameters at various VT were compared using ANOVA and Student-Newman-Keuls test. Pearson’s linear correlation was used to analyze the degree of association between variables. *P*-values less than 0.05 were considered statistically significant.

## Results

Sixty nine patients without heart and pulmonary complications undergoing selected abdominal operation were enrolled in this study. Patient characteristics were presented in Table 1. And 27 patients whose PPV values were larger than 13 % at the VT of 8 ml/kg under the initial three VT settings were considered as the subgroup.

**Table 1:** Patients’ characteristics

Variable	Global
Gender (m/f)	43/26
Age (y)	46.46 (10.43)
Height (cm)	168.20 (7.30)
Actual Body Weight (kg)	62.88 (7.56)
BMI (kg/m^2^)	22.18 (1.78)
Predicted Body Weight (kg)	62.61 (8.43)

Data are presented as mean (SD)

*BMI* body mass index

**Fig. 1:**
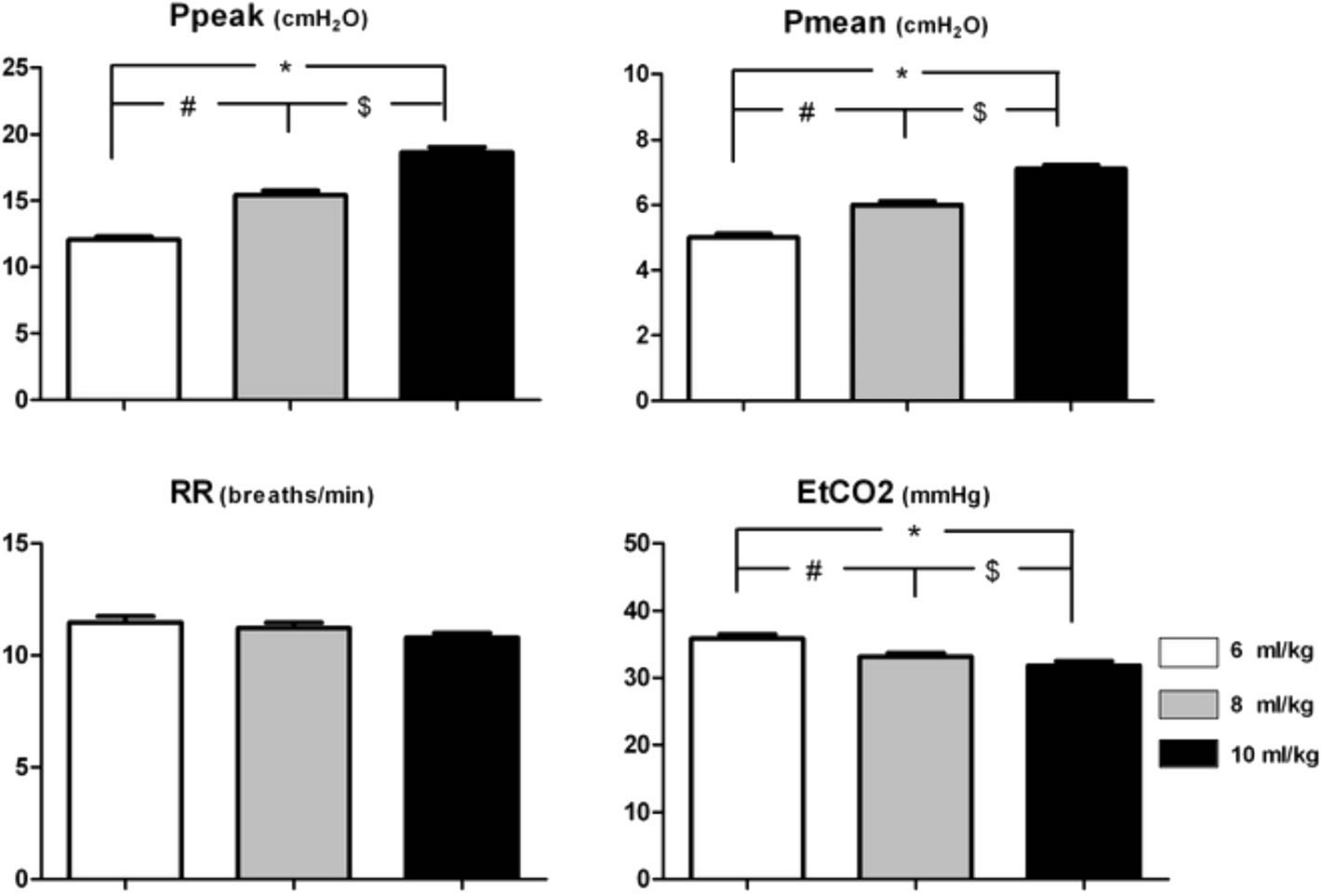
Respiratory parameters in three setting of VT. ^#^ - significant difference 8 ml/kg vs. 6 ml/kg group; ^$^ - significant difference 10 ml/kg vs. 8 ml/kg group; ^*^ - significant difference 10 ml/kg vs. 6 ml/kg group RR: respiratory rate; Ppeak: peak airway pressure; Pmean: mean airway pressure; PetCO2: End-tidal carbon dioxide partial pressure

To maintain the PetCO2 between 30 and 40 mmHg, the respiratory rate declined when the VT went up from 6 ml/kg to 8 ml/kg or 10 ml/kg. Meanwhile, the peak airway pressure and mean airway pressure increased along with VT (Fig. 1).

**Fig. 2:**
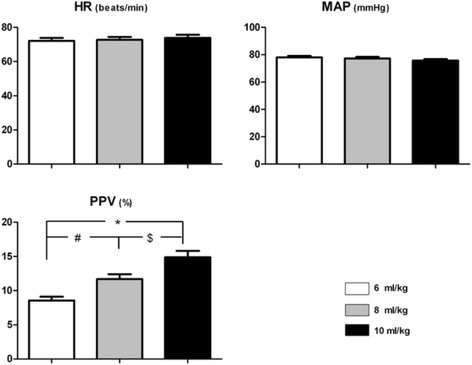
Hemodynamic parameters in three setting of VT. ^#^ - significant difference 8 ml/kg vs. 6 ml/kg group; ^$^ - significant difference 10 ml/kg vs. 8 ml/kg group; ^*^ - significant difference 10 ml/kg vs. 6 ml/kg group. HR: heart rate; MAP: mean arterial pressure; PPV: pulse pressure variation

There was no significant difference in the mean blood pressure and heart rate between different VT settings. However, PPV increased progressively when the VT changed from 6 ml/kg to 8 ml/kg or 10 ml/kg (Fig. 2).

**Fig. 3:**
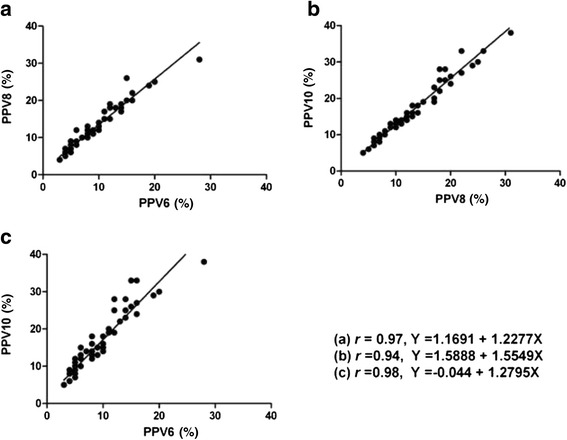
Linear relationship between PPVs under the VT of 6, 8 and 10 ml/kg before volume expansion. PPV6: pulse pressure variation at a VT of 6 ml/kg; PPV8: pulse pressure variation at a VT of 8 ml/kg; PPV10: pulse pressure variation at a VT of 10 ml/kg. Equation (**a**) represented the linear correlation between PPV6 and PPV 8. Equation (**b**) represented the linear correlation between PPV8 and PPV 10. Equation (**c**) represented the linear correlation between PPV6 and PPV10

**Fig. 4:**
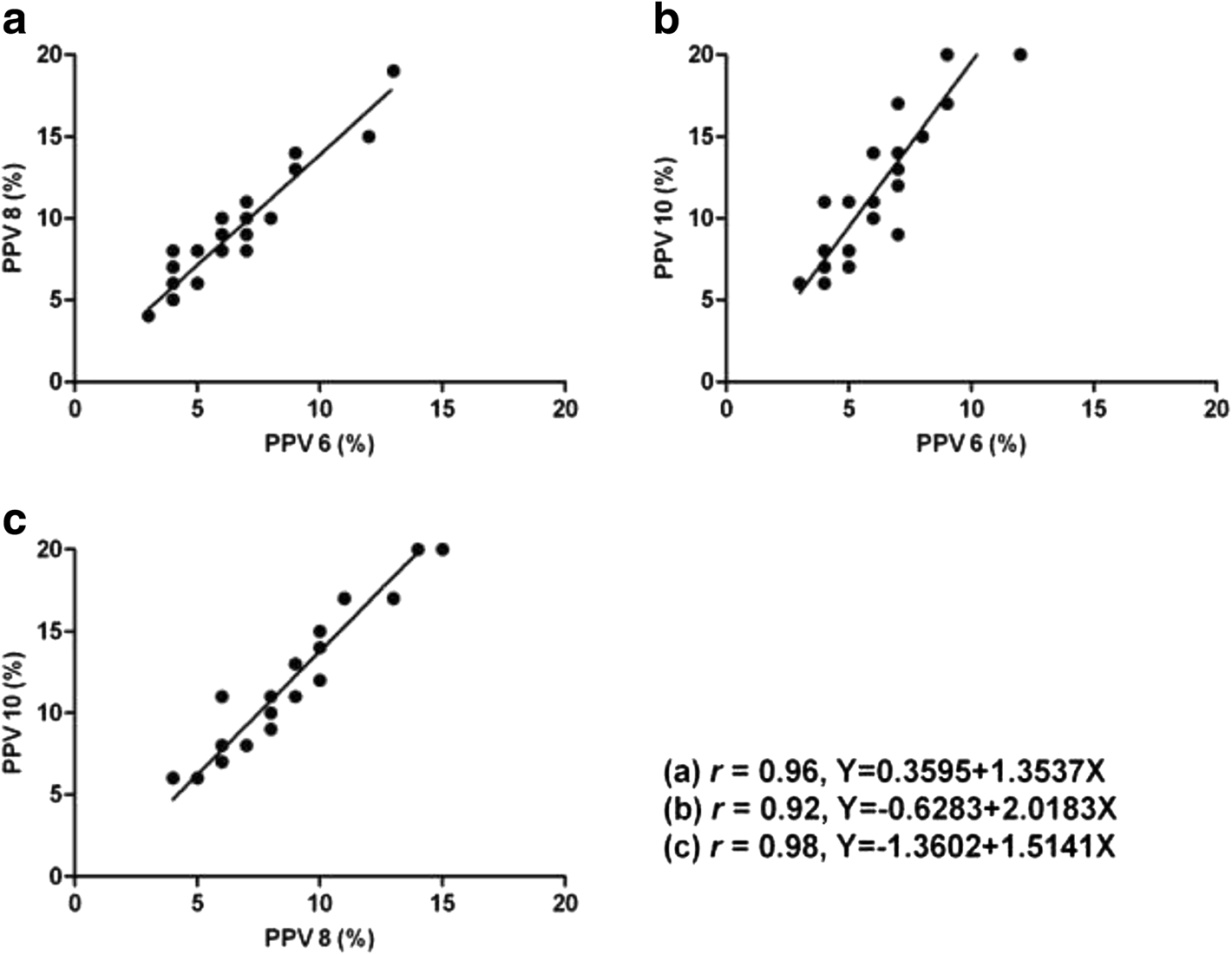
Linear relationship between PPVs under the VT of 6, 8 and 10 ml/kg after volume expansion. PPV6: pulse pressure variation at a VT of 6 ml/kg; PPV8: pulse pressure variation at a VT of 8 ml/kg; PPV10: pulse pressure variation at a VT of 10 ml/kg. Equation (**a**) represented the linear correlation between PPV6 and PPV 8. Equation (**b**) represented the linear correlation between PPV6 and PPV 10. Equation (**c**) represented the linear correlation between PPV8 and PPV10.

A significant linear correlation between PPVs at a VT of 6 ml/kg and 8 ml/kg was found in the study(*r* = 0.97), and the linear regression equation was *Y* =1.1691 + 1.2277X. There was also a significant linear correlation between PPVs at a VT of 6 ml/kg and 10 ml/kg (*r* = 0.94), and the linear regression equation was *Y* = 1.5888 + 1.5549X. Moreover, a significant linear correlation was observed between PPVs at a VT of 8 ml/kg and 10 ml/kg (*r* = 0.98; linear regression equation, *Y* = -0.044 + 1.2795X) (Fig. 3). The patients in the subgroup (27 patients who received volume expansion) showed a significant linear correlation between the PPV values under the three VT settings (PPV6 vs. PPV8: *r* = 0.96; PPV6 vs. PPV10: *r* = 0.92; PPV8 vs. PPV10: *r* = 0.98). The correlation coefficient of the subgroup was similar with the initial observations (Fig. 4).

## Discussion

The main findings of our study were that PPV increased progressively as the VT increased in the same cardiovascular status and volume status, and that there was a significant linear correlation between any two values of PPV when VT was 6, 8 or 10 ml/kg respectively.

To explore the relationship of PPVs at various VT, we enrolled patients without respiratory and cardiovascular dysfunctions in our investigation, The reason why ARDS patients were excluded was that their pathological changes might weaken the transmission of the driving pressure during mechanical ventilation [16, 17]. Another reason was clinically most of the intraoperative patients were ASA I-II without cardiopulmonary diseases. In order to avoid confounding from abdominal insufflations, abdominal retractors, interference with the sympathetic nervous system, etc, we obtained the parameters before skin incision [18–20].

Previous reports showed that tidal volume was the main factor influencing PPV during mechanical ventilation [7, 21]. For many years, a high tidal volume (10–15 ml/kg) was recommended to recruit collapsed lung tissue and improve ventilation perfusion mismatch [22, 23]. But recent studies alerted people the harm of high tidal volume ventilation and showed that low tidal volume ventilation (range of 6–8 ml/kg) may be helpful not only in the treatment of ARDS but also in the prevention of ARDS, atelectasis, and lung infections in patients without ARDS [24–28]. Therefore, the VT of 6, 8 and 10 ml/kg, which were used frequently in our clinical work, were selected in this clinical trial.

Recently, Freitas et al. found that PPV could accurately predict fluid responsiveness in septic patients ventilated at a lower VT, and there was a significant linear correlation between PPVs at a VT of 6 ml/kg and 8 ml/kg (*r* = 0.92) [14]. The similar linear correlation between PPVs in these two VT settings was also obtained in our study, but the correlation coefficient was higher (*r* = 0.97). This discrepancy could be explained by the different subjects of the two studies. In Freitas’s study, all the patients suffered from ARDS, whose mean pulmonary arterial pressure was larger than 25 mmHg; however, the enrolled patients in our study had no pulmonary inflammation or circulation system disease [14]. Moreover, we found that there was a significant linear correlation between PPVs at a VT of 6 ml/kg and 10 mL/kg (*r* = 0.94), and that the correlation was also significant between PPVs at a VT of 8 ml/kg and 10 ml/kg (*r* = 0.98). Also, based on the subgroup analysis, the high correlation coefficient of different PPV values at different VT existed no matter what the patients’ volume status was.

**Fig. 5:**
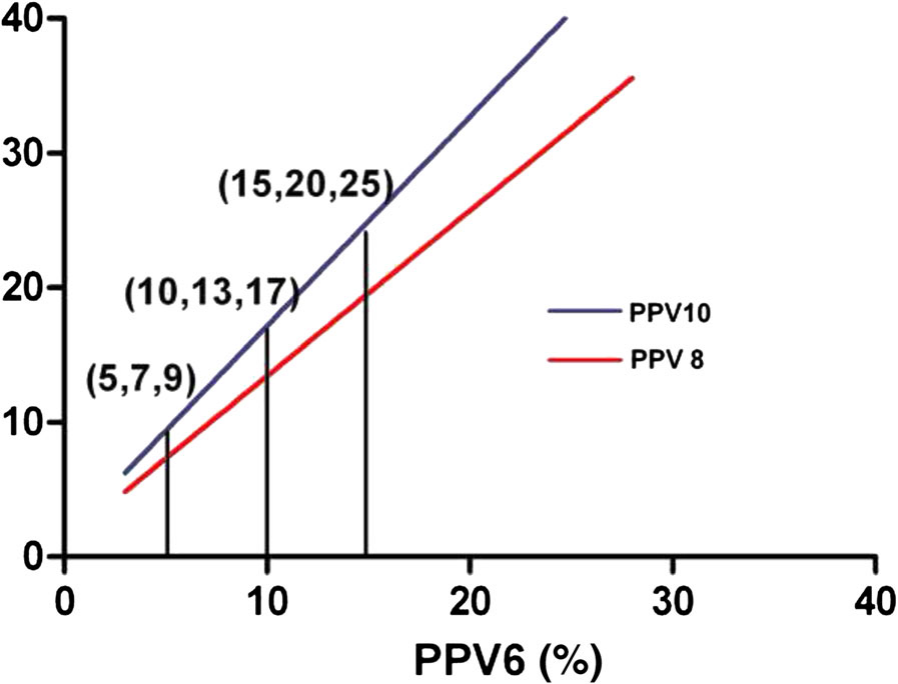
Relationship between PPVs in different VT settings. PPV6, pulse pressure variation at a VT of 6 ml/kg; PPV8, pulse pressure variation at a VT of 8 ml/kg; PPV10, pulse pressure variation at a VT of 10 ml/kg

Regressive equations could be drawn on the basis of the Pearson’s correlation analysis, (Figs. 3 and 4). In accordance to the regressive equations, the PPV at any other VT can be calculated based on the PPV obtained with the real-time monitor at a given VT. For example, if the PPV was 5 % at a VT of 6 ml/kg, based on the equation *Y* =1.1691 + 1.2277X, the PPV was 7 % at a VT of 8 ml/kg and it reached 9 % at the VT of 10 ml/kg according to the equation *Y* = 1.5888 + 1.5549X. In addition, when the values of PPV in different settings of VT were compared, we found that the bigger the PPV was, the greater the difference between any two values of PPV (Fig. 5).

Theoretically, if such regressive equations are applied in bedside monitors as a program, the values of PPV under any other VT setting in the current volume status can be calculated based on the real-time PPV value under a given VT and will be shown in real time on the monitors. It will be helpful in clarifying the volume status and thus deciding the demand of the fluid administration for patients ventilated with a low VT by deducing their PPV values under VT of 8 or 10 ml/kg.

In our study, there were some limitations to consider. The foremost was that we only assessed the relationship between the PPVs in the most commonly used VT settings (6, 8 and 10 ml/kg) in patients without cardiopulmonary disorder. Secondly, the parameters were only obtained before surgical procedure. Thirdly, the subgroup in the study only covered a portion of the initially enrolled patients. Under such circumstances, it is suggested that a series of new clinical trials be designed to evaluate the relationship between the PPV values in healthy humans under other VT settings (eg 5, 7 or 9 ml/kg) or in the patients with heart and lung diseases (eg ARDS, cardiac insufficiency, etc.). We are planning to conduct clinical trials to evaluate the relationship between the PPV values under different VT during operation, especially during some special types of surgeries like laparoscopic surgeries.

## Conclusions

In this study we have demonstrated that there was a direct linear correlation between any two values of PPV when the VT was 6, 8 or 10 ml/kg respectively. In addition, we have developed the regressive equations for the calculation of PPV values under different settings of VT. Using these regressive equations, we can calculate the PPV in another setting of VT based on the PPV at a given VT.

## Abbreviations

ARDS: Acute respiratory distress syndrome
ASA: American Society of Anesthesiologists
BIS: Bispectral index
HR: Heart rate
MAP: Mean arterial pressure
PEEP: Positive end expiratory pressure
PetCO_2_: Partial pressure of end-tidal expiration carbon dioxide
Pmean: Mean airway pressure
Ppeak: Peak airway pressure
PPV: Pulse pressure variation
RR: Respiratory rate
SVV: Stroke volume variation
VT: Tidal volume
